# Molecular cloning and functional analysis of Chinese bayberry *MrSPL4* that enhances growth and flowering in transgenic tobacco

**DOI:** 10.3389/fpls.2023.1127228

**Published:** 2023-02-01

**Authors:** Xiangqi Wu, Shuwen Zhang, Zheping Yu, Li Sun, Senmiao Liang, Xiliang Zheng, Xingjiang Qi, Haiying Ren

**Affiliations:** ^1^College of Chemistry and Life Sciences, Zhejiang Normal University, Jinhua, China; ^2^State Key Laboratory for Managing Biotic and Chemical Threats to Quality and Safety of Agro-products, Institute of Horticulture, Zhejiang Academy of Agricultural Sciences, Hangzhou, China; ^3^Biotechnology Research Institute, Xianghu Laboratory, Hangzhou, China

**Keywords:** Chinese bayberry, growth and flowering, MrSPL4, gene function, qRT-PCR

## Abstract

Chinese bayberry (*Myrica rubra*) is an important tree in South China, with its fruit being of nutritional and high economic value. In this study, early ripening (ZJ), medium ripening (BQ) and late ripening (DK) varieties were used as test materials. Young leaves of ZJ, BQ and DK in the floral bud morphological differentiation periods were selected for transcriptome sequencing to excavate earliness related genes. A total of 4,538 differentially expressed genes were detected. Based on clustering analysis and comparisons with genes reportedly related to flowering in *Arabidopsis thaliana*, 25 homologous genes were identified. Of these, one gene named *MrSPL4* was determined, with its expression down-regulated in DK but up-regulated in ZJ and BQ. *MrSPL4* contained SBP domain and the target site of miR156, and its total and CDS length were 1,664 bp and 555 bp respectively. The overexpression vector of *MrSPL4* (35S::35S::*MrSPL4*-pCambia2301-KY) was further constructed and successfully transfected into tobacco to obtain *MrSPL4*-positive plants. Based on the results of qRT-PCR, the relative expression of *MrSPL4* was up regulated by 3,862.0-5,938.4 times. Additionally, the height of *MrSPL4*-positive plants was also significantly higher than that of wild-type (WT), with the bud stage occurring 12 days earlier. Altogether, this study identified an important gene -*MrSPL4* in Chinese bayberry, which enhanced growth and flowering, which provided important theoretical basis for early-mature breeding of Chinese bayberry.

## Introduction

*Myrica rubra* (Lour.) *Sieb. et Zucc.*, of the family *Myricaceae*, is a native, economically important tree in South China where it is particularly concentrated in the south of the Yangtze River Basin (Zhang et al., 2022). Its fruit, Chinese bayberry, is not only soft, juicy and rich in flavor, but in addition to ecological benefits, it also has medical uses. Chinese bayberry is widely favored by consumers, especially in the Zhejiang Province where its fame has, to a large extent, promoted the healthy development of the Chinese bayberry industry to drive the economic development of planting areas (Jia et al., 2019; Ren et al., 2019).

The maturation period of the existing main varieties of *Myrica rubra* is about 15 days and occurs in the middle to late June. In addition, the subsequent ripening period is very short and coincides with the plum rain season in the South, which causes great harm and serious economic losses. In this context, early maturing varieties can effectively avoid the influence of plum rain, lengthen the maturity period, reduce the market pressure caused by concentrated maturation and significantly improve the economic benefits of cultivation. Therefore, the development of early maturing germplasm as well as the cultivation of new varieties with characteristics of early maturation have become important for the sustainable development of this industry. After years of observation and studies on the development of different flower buds, it was found that early flowering is an important phenotype related to early ripening. Hence, identifying flowering genes and elucidating their mechanism of action can be useful to regulate the ripening stage and create new germplasm with the improved characteristics. In this context, the SQUAMOSA promoter-binding protein-like (*SPL*) gene family is known to be important for the regulation of plant flowering, but its role in the flowering process of Chinese bayberry is yet to be reported.

The *SPL* gene family, also known as SBP protein, is a unique type of transcription factor in green plants whose members have a highly conserved SBP domain (Yang et al., 2021). The latter, which is about 80 amino acid residues in length, contains two zinc finger structures (Cys-Cys-His-Cys;Cys-Cys-Cys-His or Cys-Cys-Cys-Cys) as well as a nuclear localization signal (NLS) located at the C-terminal. Most *SPL* genes also contain highly conserved microRNA156/157 (miR156/miR157) targeting sites that regulate more complex physiological processes (Birkenbihl et al., 2005; Li et al., 2020). *SPL*s were first identified from *Antirrhinum majus* but with the rise of plant genomics, they have been isolated, identified and analyzed from a number of other plants, including *Oryza sativa* (Yang et al., 2008), *Arabidopsis thaliana* (Wu et al., 2009), *Solanum lycopersicum* (Salinas et al., 2012), *Malus × domestica Borkh.* (Li et al., 2013), *Vitis Vinifera* (Hou et al., 2013), peony (*Paeonia Suffruticosa*) (Zhu et al., 2018), *Fragaria Vesca* (Xiong et al., 2018), *Citrus sinensis* (Liu et al., 2017). Currently, *SPLs* are considered to be key genes that regulate biological processes in plants, especially since they show variations in their functions. For instance, these genes can regulate the flowering process of plants (Lei et al., 2018; Guo et al., 2019), coordinate plant root, stem and leaf development (Yu et al., 2015; Wang et al., 2018; Wang et al., 2019; Li et al., 2021), influence response to biotic and abiotic stress (Ning et al., 2017; Feyissa et al., 2019) as well as participate in secondary metabolic processes (Yang et al., 2021).

In recent years, other functions of *SPL* genes in plants have also been widely studied. For example, in *Arabidopsis thaliana*, *SPL10* was found to be highly expressed in plant leaf and root tissues, resulting in earlier flowering, narrower leaf shapes, smaller and fewer rosette leaves as well as reduced root length and root number by binding to the *AGL79* promoter (Gao et al., 2018). In addition, the expression of *AtSPL9* and *AtSPL10* in leaf primordia was also reported to affect the differentiation of apical meristem into leaves (Wang et al., 2008), while in leaf tissues, *SPL2* could control floral organs, long silique development and plant fertility by activating *AS2* (Wang et al., 2016). In rice, the GO function analysis of differentially expressed genes in blade leaves of *SPL4* mutant rice showed that *OsSPL4* gene mutations affected protein phosphorylation as well as the binding of iron ions in rice leaves, maintaining the normal plant type of rice (Hu et al., 2021). In pea (*Pisum sativum L.*), *PsSPL3a/3c* was found to be mainly expressed at the transcriptional level in leaves, hence indicating its possible involvement in leaf phase transition in the pea aging pathway (Vander Schoor et al., 2022). In maize (*Zea Mays*), *SPL4* plays an important role in bract development and meristem establishment (Chuck et al., 2010), while *SPL10/14/26* not only regulates the expression of *ZmWOX3A* and auxin related genes but is also involved in the development of epidermal hair on maize leaves (Kong et al., 2021). Finally, an analysis of the expression of *MdSBP* genes in apple leaves after different hormone treatments showed that many of the genes responded to different plant hormones, thereby suggesting that *MdSBP* genes could be involved in response to hormone signals during stress or apple development (Li et al., 2013).

Therefore, based on the previous genome sequencing of *Myrica rubra* (Ren et al., 2019) the latter’s *SPL* gene family was identified and analyzed based on bioinformatics methods. This was followed by the cloning of an *SPL* gene and its subsequent heterologous expression in *Nicotiana benthamiana* L. by constructing an overexpression vector to validate the functions of the gene. Altogether, this study is expected to provide a theoretical basis for revealing the regulatory pathway of flowering in Chinese bayberry.

## Materials and methods

### Material information

By referring to the expression of genes related to male and female flowering as described by Jia et al. (2019), three experimental materials with different flowering stages were selected from the Lanxi International Chinese bayberry Research Center(Latitude 29.30°N, longitude 119.60°E) in November 2019 (period during which floral buds can be morphologically differentiated). These included the early maturing variety “Zaojia” (ZJ), the medium maturing variety “Biqizhong” (BQ) and the late maturing variety “Dongkui” (DK) (**Table 1**). The ages of all selected trees were around 15 years, and they were in consistent cultivation conditions. Each variety was sampled in triplicates, and for transcriptome sequencing, young leaves (not unfolded) were taken from annual branches facing south and at 1 m above the ground.

**Table 1:** Phenological periods of different test materials.

Test material	No.	Flower bud formation time	First flowering period (month-day)	Maturity period (month-day)
Zaojia	ZJ	Mid-November	April 1st	June 6th
Biqizhong	BQ	Mid-December	April 4th	June 15th
Dongkui	DK	Mid-January	April 10th	June 25th

### Transcriptome sequencing and screening of differentially expressed genes

A polysaccharide polyphenol RNA extraction kit was used for extracting total RNA from the samples (TIANGEN, Beijing), and after RNA detection, Biomarker Biotechnology Co., Ltd. was commissioned to carry out the transcriptome sequencing. For this purpose, magnetic beads with Oligo (dT) were used to enrich the total RNA of the samples before fragmenting the mRNA with the fragmentation buffer. The first cDNA strand was then synthesized with random hexamers using mRNA as template, and this was followed by the synthesis of the second cDNA strand by adding buffer, dNTPs, RNase H and DNA polymerase I. After purification with the QiaQuick PCR kit and elution with EB buffer, end repair was performed, poly (A) tails were added and sequencing adaptors were connected. Appropriate fragments were then selected by gel electrophoresis prior to PCR-based amplification. The resulting libraries were eventually sequenced on an Illumina HiSeq4000.

After gene splicing, protein sequences were aligned with those from eight public databases (COG, GO, KEGG, KOG, Pfam, Swissport, eggNOG and Nr) using a threshold of e≤e^-10^. The BLAST algorithm was then used for sequence similarity comparison, with the resulting sequence similarities subsequently used for functional annotations. Relative gene expression was assessed based on RPKM (Reads Per Kilobase of exon model per Million mapped reads) where larger RPKM values were indicative of higher expression levels (Trapnell et al., 2010).

Differentially expressed genes were screened by the false discovery rate (FDR) (Zhao et al., 2020), with a |log_2_ fold change|≥2 and an FDR<0.5 selected as thresholds for a gene to be considered as being differentially expressed.

### SPL gene family analysis

The *SPLs* of Chinese bayberry were isolated and identified by tBLASTn analysis of AtSPL amino acid sequences obtained from the genomic data of Chinese bayberry (Ren et al., 2019). The Chinese bayberry *SPLs* and target sites of miRNA156 were then predicted and confirmed using Genscan Web (http://genes.mit.edu/GENSCAN.html) as well as the BLASTx algorithm (http://www.ncbi.nlm.nih.gov/BLAST). After obtaining the nucleotide and amino acid sequences of 16 Arabidopsis and 46 apple *SPL* family genes from the Plant Transcription Factor Database (PlnTFDB3.0) (http://plntfdb.bio.uni-potsdam.de/v3.0/), phylogenetic trees were also constructed using the NJ method in MEGA 7.0, along with full-length protein sequences and the test parameter (bootstrap) set to 1000. The exon and intron structures of Chinese bayberry *SPL* genes were obtained by Gene Structure Display Sever (http://gsds.cbi.pku.edu.cn/index.php).

### Strains and vectors

*Escherichia coli* competent cell DH-5α (Shanghai Jinchao Technology Development Co., LTD.), *Agrobacterium tumefaciens* strain GV3101 and pCambia2301-KY vectors (Shanghai Kaiyi Biotechnology Co., LTD.) were the main requirements of the study.

### Primer design and gene cloning

Using the genome sequence of Chinese bayberry (Ren et al., 2019), specific primers for both sides of the open reading framework (ORF) of the target gene were designed with Primer Premier 5.0 software for gene cloning. Total RNA extraction was also performed on healthy Chinese bayberry leaves using the modified cetyl trimethyl ammonium bromide (CTAB) method, with the extracted RNA acting as template to synthesize cDNA according to the instructions of the HiScript 1st Strand cDNA Synthesis Kit (Vazyme). This was followed by PCR amplification with the Phanta Max ultra-fidelity DNA polymerase (Vazyme), using the cDNA as template. In this case, each reaction consisted of the following component: 1 μL of Phanta Max super-Fidelity DNA Polymease, 2 μL of cDNA, 2 μL each of both forward and reverse primers, 25 μL of 2×Phanta Max Buffer, 1 μL of dNTP Mix and ddH_2_O (for making up the volume to 50 μL), while the PCR procedure involved an annealing temperature of 49 °C and an extension rate of 1 kb/min, carried out for 39 cycles. Other operations shall follow the product instructions of Vazyme Company. The amplified products were finally detected on 1.5% agarose gel, before being sent to the company for sequencing to verify the accuracy of cloning results.

### Construction of an overexpression vector and Agrobacterium transformation

The restriction enzyme *BamHI* (Takara Company) was first used to linearize the vector before extracting the pCambia2301-KY plasmid for digestion with the same enzyme. The overexpression vector was then constructed with Vazyme recombinant enzyme at 37 °C by using the following components: 2 μL of 5×CE II Buffer, 1 μL of Exnase II, 4 μL of linearized carrier, 1 μL of insert fragment and ddH_2_O (to make up the volume to 10 μL). After 30 min of reaction, the vector was placed on ice for cooling. The cells were then transfected into competent *E. coli* DH-5α cells and cultured in LB medium containing 50 mg/L Kan. This enabled the selection and subsequent culture of resistant colonies for the positive detection of the gene by PCR. The amplified products were finally sent for sequencing. The positive transformer colony plasmid was extracted and transfected into *Agrobacterium tumefaciens* GV3101 and sterile glycerol was added to preserve the bacteria at -80 °C until required for the next transfection.

### *Agrobacterium tumefaciens*-mediated transfection of tobacco

Tobacco Benn was selected for this set of transformation experiment. WT tobacco was infected with Agrobacterium carrying recombinant vector plasmids of target genes using the leaf disk method. After four times of continuous screening/subculture, resistant buds were eventually recovered and transferred to a rooting medium to induce roots. Once the root system was vigorous, healthy and completely regenerated plants were transplanted to the soil (nutrient soil-vermiculite ratio was 1:1 or 2:1) where they were maintained until the T_0_ generation for seed collection.

Collected seeds were sterilized with 70% ethanol, 30% sodium hypochlorite or 40% of 84 disinfectant and rinsed with sterile water 5-6 times. Seeds were then added to 1/2 MS solid selective medium containing 80 mg/L of Kan, and vernalized at 4°C for 2 days to break dormancy. They were subsequently cultured in a light incubator of the laboratory of Zhejiang Academy of Agricultural Sciences (light 28°C, 16 h, Darkness 25°C, 8 h, humidity 50%-70%). After about a week, the seeds were transferred to the soil to maintain grow. The leaves of the transgenic resistant plants and the wild-type ones of the T_1_ generation were randomly sampled and stored at -80°C after being frozen in liquid nitrogen.

### Determination of relative gene expression

Transgenic positive plants of T_1_ generation were obtained through screening with 80 mg/L Kan, 1/2 MS solid selective medium and PCR. The leaves of grown plants were collected, and total RNA was extracted with the RNA simple Total RNA Kit (TIANGEN) after quick-freezing in liquid nitrogen. In addition, synthesis reactions were also performed in 10-μL reaction volumes with the first Strand cDNA synthesis kit. For this purpose, the following components were used as required by the FastFire qPCR PreMix (SYBR Green) Kit (TIANGEN): 5 μL of 2×FastFire qPCR PreMix, 1 μL of forward primer and reverse primers (10 μm) and 1 μL of cDNA template. The reaction was performed on a Light Cycler 96 real-time PCR instrument under the following conditions: 95°C for 60 s, followed by 45 cycles, each at 95°C for 5 s, 63°C for 10 s and 72°C 15 s. Three technical replicates were set for each sample. Quantitative primers were designed according to gene sequences, with *Ntactin*-F/R selected as the reference gene (Zhao et al., 2020), and WT tobacco acting as the control to determine the relative expression of target genes. The 2^-△△Ct^ method was used to process the data (Livak and Schmittgen, 2001), while the IBM SPSS Statistics 22 and Origin 2022/Microsoft Excel 2010 were used for statistical analysis and plotting respectively.

### Cloning, structural analysis and construction of overexpression vector of *MrSPL4*

Primers were therefore designed based on the reference genome sequence (**Table S1**), with ORF sequences of the *MrSPL4* gene in ZJ, BQ and DK subsequently obtained by PCR amplification. Therefore, it was inferred that the expression of this gene was different between the different test materials, probably due to the promoter element, but this remained to be experimentally verified. The full length and CDS of *MrSPL4* were 1,664 bp and 555 bp respectively. The amplified product was first recovered, and the vector was digested with *BamHI*. The resulting enzymatically digested product was then recombined with the amplified product to construct a plant overexpression vector. The latter was transformed into *E. coli* competent cells DH-5α before identifying the transformed bacterial solution by PCR.

### Verification of *MrSPL4* positive tobacco plants

*Agrobacterium*-mediated transformation of *Nicotiana benthamiana* with the recombinant plasmid 35S::*MrSPL4*-pCambia2301-KY was performed. The tobacco leaves infected by *Agrobacterium tumefaciens* were directly transferred to a selective medium containing kanamycin (Kan) to induce differentiation and budding. After the buds had grown to 2-3cm, they were inserted into a rooting medium to induce the formation of roots. Once the root system was vigorous, the seedlings were then tempered and transplanted to soil for culture to obtain completely regenerated tobacco with Kan resistance. Leaf DNA from the resistant regenerated tobacco plants to be tested was used as a template for PCR-based validation.

## Results

### Evaluation of transcriptome data of young leaves from different flowering materials

The transcriptome sequencing of nine samples of young leaves (three biological replicates for each variety) in the floral bud morphological differentiation period was completed, and a total of 59.78 Gb of clean data, with an average GC content of 47.28% and a Q30 base ratio of 93.55%, were obtained. After comparison with the reference genome (Ren et al., 2019), the percentage of clean reads aligned to the reference genome was found to be 95.39% (**Table S2**, the transcriptome data of BQ and DK were uploaded to https://bigd.big.ac.cn/gsa/browse/CRA008253, and the datasets of ZJ generated and analyzed during the current study are available in the NCBI repository https://www.ncbi.nlm.nih.gov/sra/PRJNA733585). The number of differentially expressed genes between the three samples was then compared. In this case, 623 genes were differentially expressed between ZJ and DK, and of these, 476 were up-regulated and 147 were down-regulated. Similarly, 2,343 genes were differentially expressed between ZJ and BQ, with 1,385 and 958 genes being up-regulated and down-regulated respectively. Finally, the number of differentially expressed genes between DK and BQ was 1,572, with 734 and 838 being up-regulated and down-regulated respectively (**Table 2**).

**Table 2:** Differentially expressed genes between pairs of samples.

DEG Set	DEG Number	Up-regulated	Down-regulated
BQ Vs DK	1,572	734	838
ZJ Vs BQ	2,343	1,385	958
DK Vs ZJ	623	476	147

### KEGG analysis of differentially expressed genes

**Figure 1:**
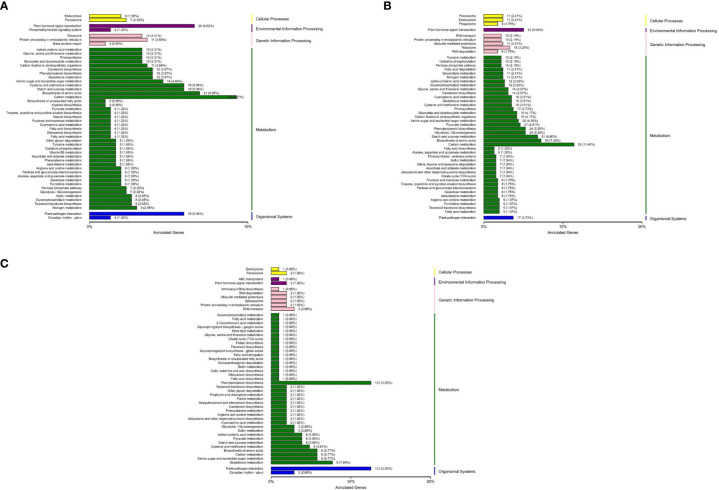
KEGG classification of differentially expressed genes in the different test materials. **(A)** KEGG classification of differentially expressed genes between BQ and DK. **(B)** KEGG classification of differentially expressed genes between BQ and ZJ. **(C)** KEGG classification of differentially expressed genes between DK and ZJ.

Through KEGG enrichment analysis, it was found that the differentially expressed genes mainly involved functions such as cellular processes, environmental information processing, genetic information processing, metabolism and organic systems (**Figures 1A–C**). The pathways that were significantly enriched in all the three groups included phytohormone signal transduction, sulfur and carbon metabolism, fatty acid, phenylpropionic acid, pyruvic acid, α-linolenic acid metabolism, glycine, serine, threonine, arginine, proline, cyano amino acids, cysteine and methionine metabolism, terpenoid skeleton biology, carotenoid biosynthesis, glutathione and glycerophosphatide metabolism, glycolysis/gluconeogenesis, starch and sucrose, amino sugar and nucleotide sugar metabolism, protein processing in the endoplasmic reticulum. The results indicated that these pathways may participate in the regulation network of Chinese bayberry flowering or other important pathways.

### Identification of *MrSPL4* based on flowering-related differentially expressed genes

**Figure 2:**
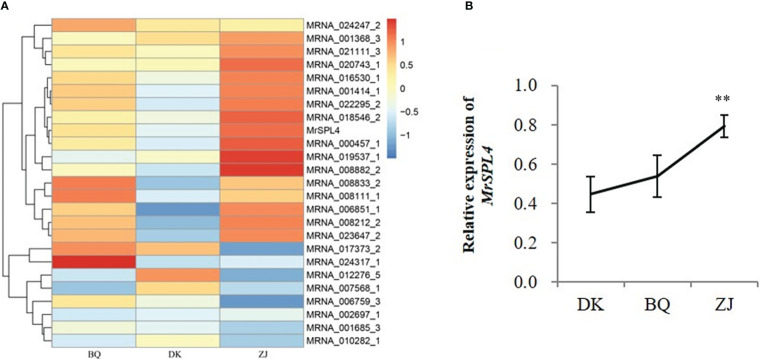
Expression of flowering-related differential genes. **(A)** The expression heat map of flowering related genes. **(B)** The relative expression of *MrSPL4* in different samples. Values are the mean ( ± SD) of three replicates, ** indicates 0.001< *p <*0.05 by the Student’s t-test.

The differentially expressed genes mentioned above were compared with 306 flowering genes reported in *Arabidopsis* (Bouché et al., 2016) and 25 genes were found to be homologous in Chinese bayberry (**Figure 2A**). In particular, one of the differentially expressed genes, MRNA_003335_1, was down-regulated in DK but up-regulated in ZJ and BQ. This gene also contained the *SBP* domain and belonged to the *SPL* gene family, named *MrSPL4*. The relative expression of *MrSPL4* was therefore verified by qRT-PCR (**Figure 2B**), and the results showed that ZJ had the highest expression, followed by BQ, with DK showing the lowest expression level. These results were, in fact, consistent with the expression determined by the transcriptome.

### Gene analysis of *SPL*s gene family in Chinese bayberry

**Figure 3:**
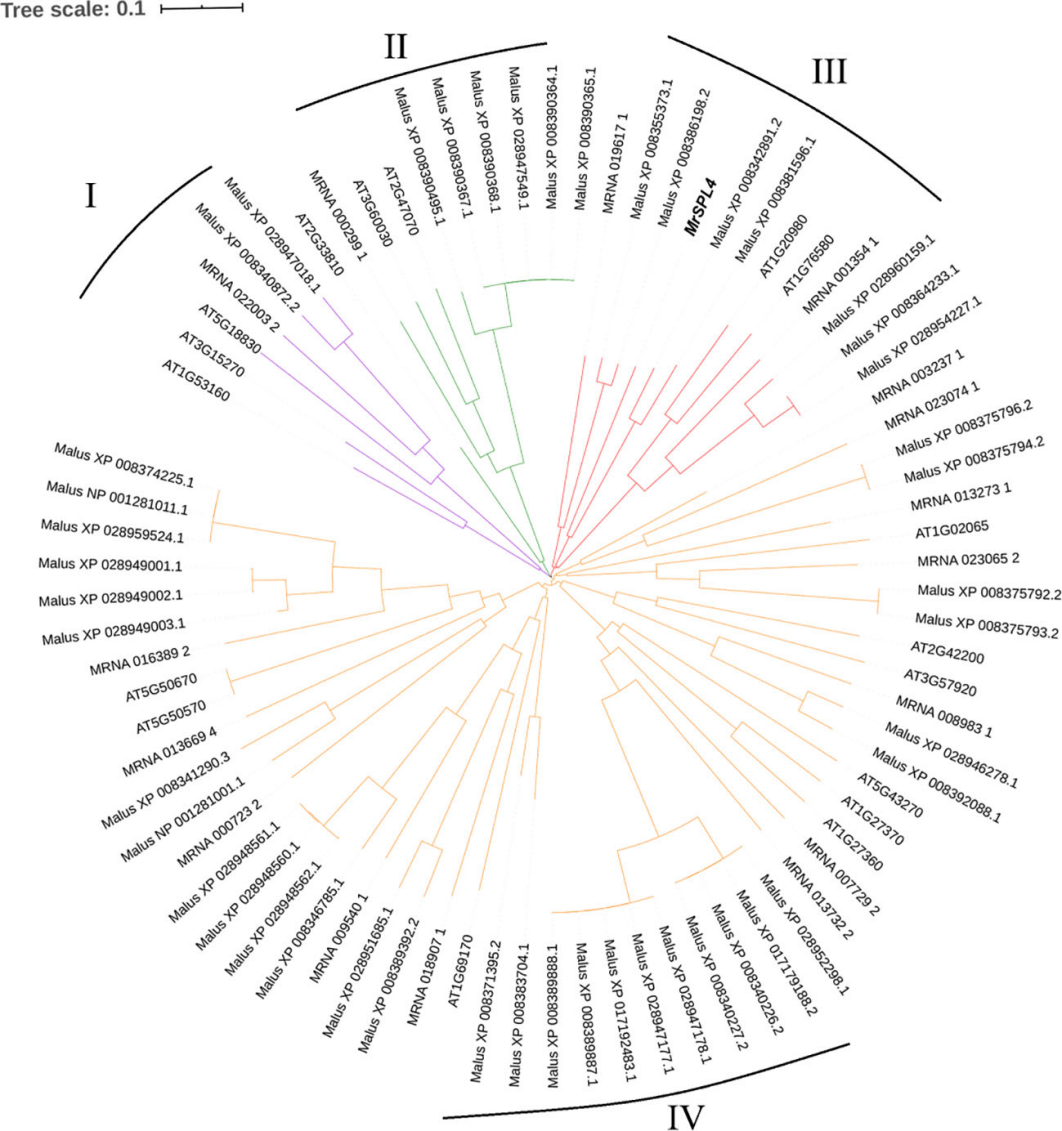
Evolutionary analysis of the SPL family gene in *Myrica rubra* and other species.

Through the screening of all genes in the reported genome provided in Ren et al. (2019), 17 *SPL* family genes with *SBP* domains were found in the Chinese bayberry genome (**Table 3**). Of these, 12 genes including *MrSPL4*, contained the target site of miR156 in the CD region. The software MEGA7.0 was then used to analyze the evolution of 17 *SPL* genes in *Myrica rubra* (*MrSPL*), 16 *SPL* genes in *Arabidopsis thaliana* and 46 *SPL* genes in apple (**Figure 3**). In this case, it was observed that the *SPL* proteins could be divided into four different groups (I, II, III and IV), with each containing at least one *MrSPL* gene. More specifically, groups I and II contained one *MrSPL* each, group IV contained twelve *MrSPLs* and group III contained three Chinese bayberry *SPL* genes, including *MrSPL4*. *MrSPL4* had the highest homology with the AT1G20980 (*AtSPL14*) gene in *Arabidopsis thaliana*, with previous studies showing that this gene (*AtSPL14*) not only promoted the normal growth and development of *Arabidopsis thaliana*, but also played a crucial role in the development of flowering as well as the transformation from a vegetative to reproductive growth. Thus, it was speculated that *MrSPL4* could be playing an important role in the flowering process of Chinese bayberry.

**Table 3:** Gene status of the SPL family in *Myrica rubra* genome.

Gene ID	Conserved domain	Target site of miR156
MRNA_003237_1	*SBP* domain	No
MRNA_013273_1	*SBP* domain	No
MRNA_013732_2	*SBP* domain	Yes
***MrSPL4***	*SBP* domain	Yes
MRNA_019617_1	*SBP* domain	No
MRNA_009540_1	*SBP* domain	Yes
MRNA_018907_1	*SBP* domain	Yes
MRNA_022003_2	*SBP* domain	Yes
MRNA_023074_1	*SBP* domain	Yes
MRNA_023065_2	*SBP* domain	No
MRNA_000299_1	*SBP* domain	No
MRNA_007729_2	*SBP* domain	Yes
MRNA_013669_4	*SBP* domain	Yes
MRNA_016389_2	*SBP* domain	Yes
MRNA_001354_1	*SBP* domain	Yes
MRNA_008983_1	*SBP* domain	Yes
MRNA_000723_2	*SBP* domain	Yes

### Sequence alignment and overexpression vector construction of *MrSPL4*

**Figure 4:**
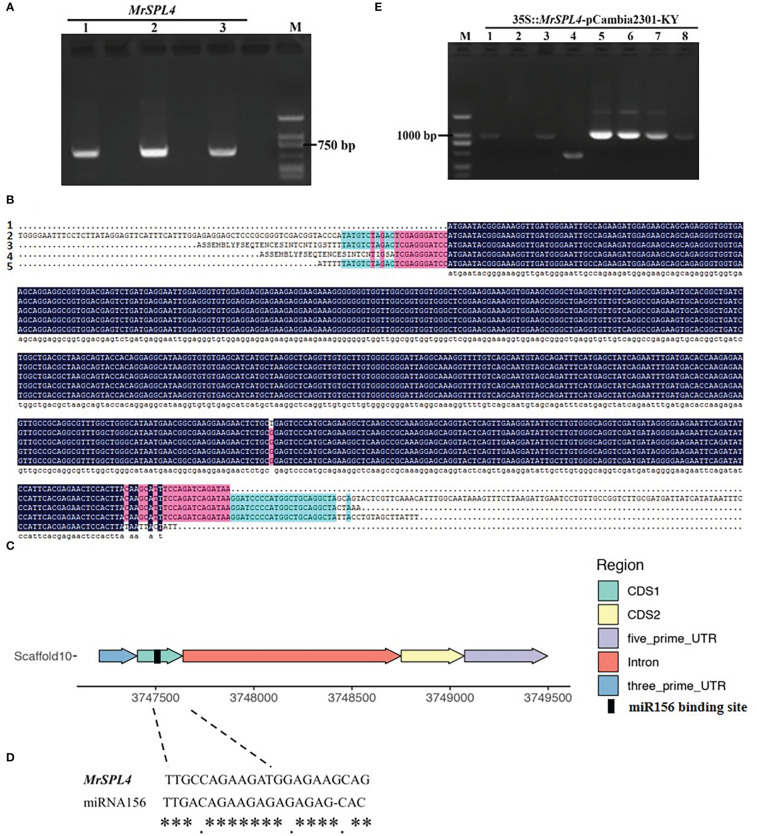
Cloning, sequence alignment, structure, miRNA156 target and construction of overexpression vector of *MrSPL4* gene in different test materials. **(A)** The result of the coding region of *MrSPL4* electrophoresis, M: DL2000 DNA marker, 1-3: *MrSPL4* gene cloning in different samples. **(B)** The CDS sequence of *MrSPL4*, 1 refers to genome sequence, and 2, 3, 4 and 5 refer to gene sequence in ZJ, ZJ, BQ and DK, respectively. **(C)** The gene structure of *MrSPL4*. **(D)** The binding site of *miRNA156*. ** represent the consistency of the binding site. **(E)** The PCR identifying result of the transformed bacterial solution, M: DL2000 DNA marker, 1-8: 35S::*MrSPL4*-pCambia2301-KY.

The electrophoresis results (**Figure 4A**) showed that the bands were consistent with the expected amplification product size, hence indicating that the sequence of the coding region of *MrSPL4* was successfully obtained. No differences in the gene sequence were noted between the three samples (**Figure 4B**). *MrSPL4* also contained two exons and one intron (**Figure 4C**), along with a binding site of *miRNA156* in the CDS1 region (**Figure 4D**).

The overexpression vector of *MrSPL4* was constructed and transformed into *E. coli* competent cells DH-5α before identifying the transformed bacterial solution by PCR (**Figure 4E**). The results showed that bands 1, 3, 5, 6, 7 and 8 were consistent with the expected target fragments. In fact, preliminary results further showed that the *MrSPL4* gene was successfully inserted into the vector to yield six positive transformant colonies. Two of these positive colonies were randomly selected for sequencing, with the results being still consistent. This experiment therefore showed that the overexpression vector of *MrSPL4* gene was successfully constructed and named as 35S::*MrSPL4*-pCambia2301-KY.

### Regeneration and identification of *MrSPL4*-positive tobacco plants

**Figure 5:**
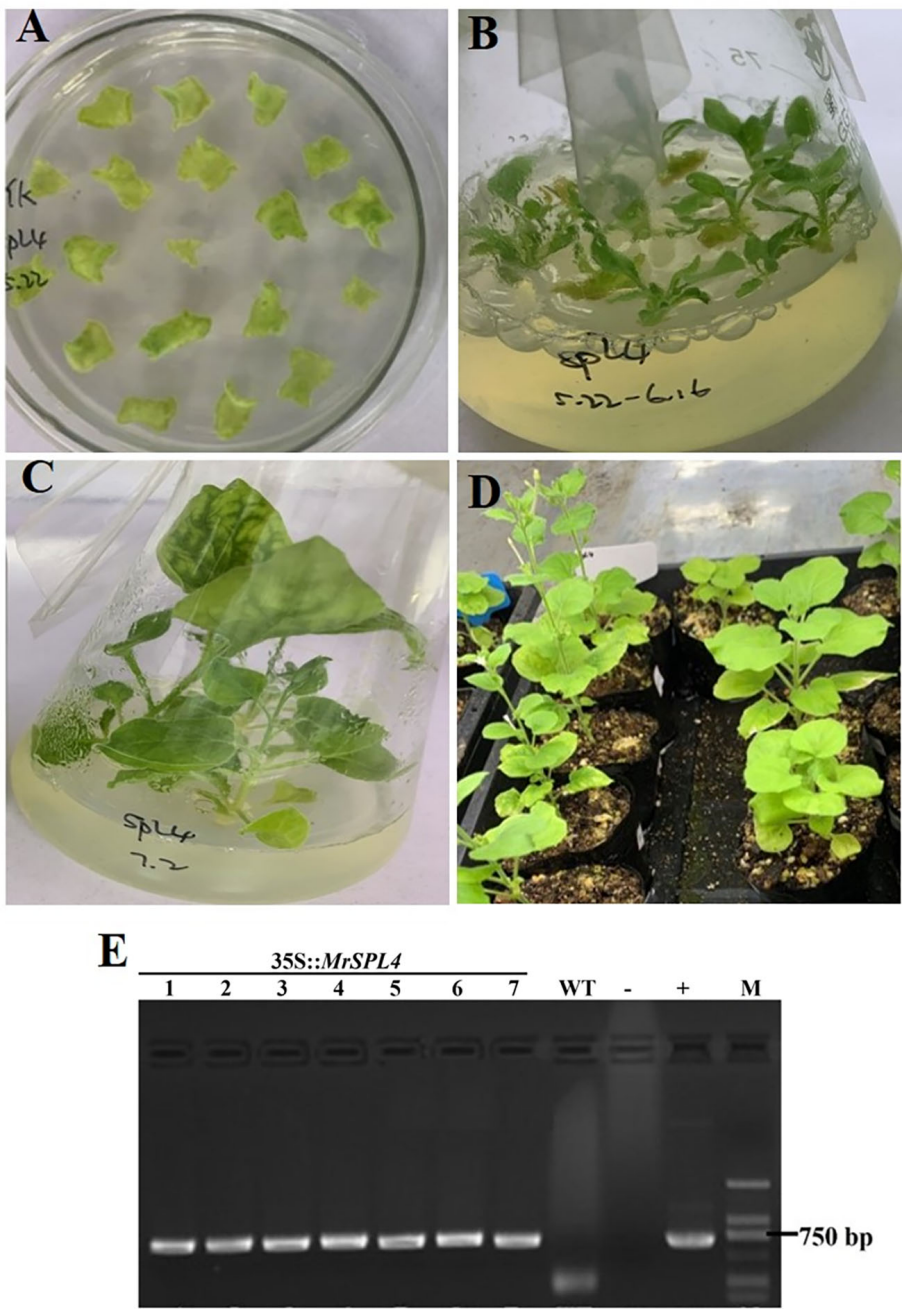
Regeneration of positive tobacco plants and PCR identification of Kan resistance. **(A)** Kanamycin medium for bud differentiation. **(B)** Resistant plant culture. **(C)** Rooting of resistant plants. **(D)** Transplanted seedling culture. **(E)** Transgenic tobacco identification using genomic PCR, 1~7: resistant regenerated tobacco, W: negative control, -: blank control, +: positive control, M: DL2000 DNA marker.

The tobacco leaves infected by *Agrobacterium tumefaciens* were induced differentiation and budding (**Figure 5A**), and small seedlings were further grown and induced roots (**Figures 5B, C**). The regenerated tobacco with Kan resistance was finally transplanted to soil (**Figure 5D**). Additionally, *MrSPL4*-F and *MrSPL4*-R were used as primers, water was set as a blank control, the 35S::*MrSPL4*-pCambia2301-KY expression vector plasmid was used as a positive control and leaf DNA of WT plants was used as a negative control. The results (**Figure 5E**) showed that for all resistant regenerated tobacco, the target band was amplified, with its size being consistent with that of the positive control. In addition, no specific bands were observed in WT tobacco and the blank control. Hence, the results showed that the *MrSPL4* gene was successfully transferred into tobacco.

### Relative expression and phenotype of *MrSPL4* in positive tobacco plants

**Figure 6:**
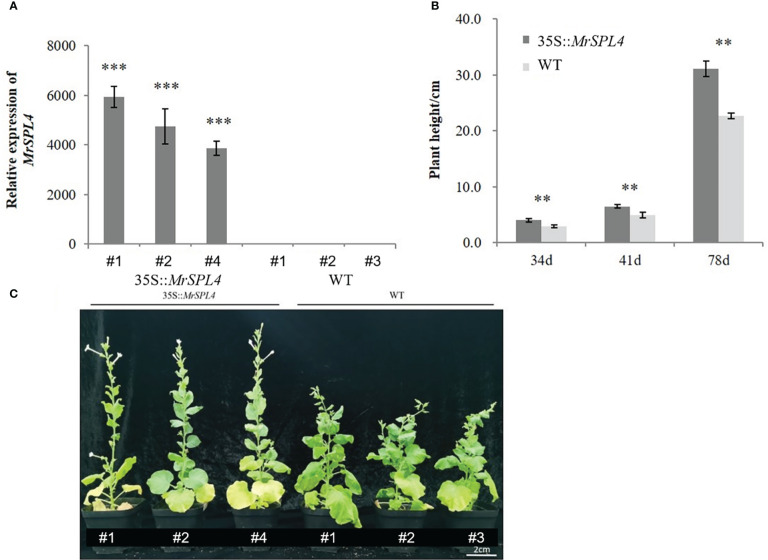
Analysis in the T_1_ generation of positive tobacco lines. **(A)** *MrSPL4* expression in positive and WT tobacco. **(B)** The plant height of positive and WT tobacco. 34d, 41d and 78d indicate the number of days after transplanting. **(C)** The phenotype of positive and WT tobacco. Values are the mean ( ± SD) of three replicates, ** indicates 0.001< *p <*0.05, *** indicates *p <*0.001 by the Student’s t-test.

Three positive tobacco lines (35S::*MrSPL4*) from the T_1_ generation were randomly selected to detect the expression level of the *MrSPL4* gene. Results showed that the relative expression level was significantly higher than that of WT tobacco, with the up-regulation multiple being between 3,862.0-5,938.4 (**Figure 6A**). This was a clear indication that the gene was overexpressed in transformed tobacco.

The plant heights for 35S::*MrSPL4*-transformed tobacco and WT ones were measured on 34 d, 41 d and 78 d after transplanting and found to be significantly higher for the transformed plants compared with the WT (**Figure 6B**). Furthermore, the growth rate was also significantly faster than for the WT. In terms of the budding stages, those of 35S::*MrSPL4*-transformed tobacco and WT plants were 34 days and 46 days after transplanting, respectively. Based on the above, it could be concluded that 35S::*MrSPL4*-transformed tobacco showed characteristics of rapid plant growth and early flowering (**Figure 6C**). Therefore, it was speculated that *MrSPL4* gene affected the phenotype of transgenic tobacco to promote plant growth and flowering.

## Discussion

As a specific and important transcription factor in plants, the *SPL* gene family has a highly conserved SBP domain which plays an important regulatory role in plant growth and development. Although the *SPL* gene family has been widely isolated and identified in many plants such as Arabidopsis and rice, research on its role in *Myrica rubra*, an economically important fruit in South China, has not been reported. Previous studies (Yang et al., 2008) have shown that the number of *SPL* gene family members varies in different species, thereby leading to the diversification of gene functions and this was confirmed in the current study. In addition, 17 *MrSPL* gene family members with SBP domains were identified in the genome of *Myrica rubra*, with this number being close to that of *SPL* gene family members in *Arabidopsis* (Wu et al., 2009) and tomato (Cui et al., 2020), but greatly different from that of *Gossypium hirsutum* L (Cai et al., 2018). and apple (Li et al., 2013). In general, members in the same subgroup are likely to have the same or quite similar functions. For example, *AtSPL2*, *AtSPL10* and *AtSPL11* inhibit root growth, while other members of this group, *CsSPL2* and *CsSPL10*, also participate in the regulation of root development (Yang et al., 2021). In the present study, phylogenetic-based analyses showed that *SPLs* could be divided into four groups, with each containing at least one *MrSPL* gene. Since *MrSPL4* was found to be homologous with the *AtSPL14* gene, it was therefore speculated that *MrSPL4* could be playing a similarly important role in plant growth and development, although it is likely that the gene could also have different functions.

Previous studies have found that flowering is an important sign of plant growth and development, and consequently, research on the role of the *SPL* gene family in the regulation of plant flowering has attracted significant interest. For example, *AtSPL3*/*4*/*5* participates in the photoperiod and the age pathway, and as such, it can promote the early flowering of *Arabidopsis* by upregulating the expression of downstream genes (Hyun et al., 2016). Similarly, overexpression of the *EjSPL3/4/5/9* genes in loquat causes transgenic *Arabidopsis thaliana* to exhibit characteristics of early flowering (Jiang et al., 2019), while strawberry *FvSPL10-OE* plants were shown to bloom 3-5 days earlier (Xiong et al., 2019). Despite the above observations, the functions of *MrSPL4* in the flowering process of Chinese bayberry remains unknown. In order to verify its role, the gene was cloned from Chinese bayberry to yield transgenic tobacco overexpressing *MrSPL4*. In this study, the relative expression of *MrSPL4* positive tobacco plants was significantly increased by 3,862.0-5,938.4 times compared with WT under long sunshine conditions. Moreover, the plant heights of transformed tobacco plants were significantly higher than WT tobacco, with the budding period also occurring 12 days earlier. This indicated that the *MrSPL4* gene responded to the flowering process of transgenic tobacco, showing early flowering and increased plant height. In addition, the current study found that the sequence of the *MrSPL4* gene in different Chinese bayberry varieties had no differences, although its expression level did differ in different Chinese bayberry varieties. It was speculated that these differences could be linked to promoter elements but this would need follow-up experiments for validation.

To sum up, 17 members of the *SPL* gene family with SBP domains were identified in *Myrica rubra*. Of these, the *MrSPL4* gene was isolated, cloned and verified in tobacco. The results showed that *MrSPL4* could regulate the flowering process of plants, accelerate their growth and endow the plants with early flowering phenotypes, thus supporting the view that this gene exerted multiple regulatory functions on plant growth and development. These results also provide a basis for further elucidating *MrSPL4*’s regulatory mechanism for flowering in *Myrica rubra* in order to achieve genetic improvement and gene breeding of this plant in the future. Therefore, the *MrSPL4* gene needs to be further studied, especially with regards to its promoter region.

## Data availability statement

The datasets presented in this study can be found in online repositories. A link to the data can be found below: https://bigd.big.ac.cn/gsa/browse/CRA008253.

## Author contributions

XW, ZY, and SZ performed the experiments. XQ assisted with design of the project. LS, SL, XZ, and HR assisted with the primary data analysis. XW and SZ wrote the manuscript. All authors contributed to the article and approved the submitted version.
