# Supplementary material for: Molecular cloning and functional analysis of Chinese bayberry MrSPL4 that enhances growth and flowering in transgenic tobacco
Source: Front Plant Sci. 2023 Feb 1;14:1127228. doi: 10.3389/fpls.2023.1127228 (PMC9929451; doi:10.3389/fpls.2023.1127228)
Supplement: Supplementary file 1 [file Table_1.docx]

**Supplementary materials**

**TABLE S1** Primer sequences

| Primer name | Primer sequence (5' to 3') |
| --- | --- |
| *MrSPL4*-F | ATGTCTAGACTCGAGGGATCCATGAATACGGGAAAGGTTGATG |
| *MrSPL4*-R | AGCCTGCAGCCATGGGGATCCTTATCTGATCTGGAAATGCTTG |
| Q-*MrSPL4*-F | GCATAATGAACGGCGAAGG |
| Q-*MrSPL4*-R | ATCTGGAAATGCTTGTAAGTGGA |
| Q-*NtActin*-F | CTATTCTCCGCTTTGGACTTGGCA |
| Q-*NtActin*-R | ACCTGCTGGAAGGTGCTGAGGGAA |

**TABLE S2** Statistics of sequencing data and alignment results

| Samples | Clean reads | Clean bases (bp) | GC Content (%) | ≥Q30 (%) | Mapped Reads Ratio (%) |
| --- | --- | --- | --- | --- | --- |
| BQ-1 | 21,403,050 | 6,390,283,060 | 47.35 | 92.68 | 95.32 |
| BQ-2 | 20,776,437 | 6,193,566,414 | 47.38 | 93.55 | 95.82 |
| BQ-3 | 27,227,360 | 8,115,985,288 | 47.30 | 93.79 | 95.68 |
| ZJ-1 | 20,138,974 | 6,004,597,524 | 47.07 | 93.77 | 94.96 |
| ZJ-2 | 20,316,870 | 6,058,667,134 | 47.77 | 93.22 | 95.98 |
| ZJ-3 | 21,634,935 | 6,455,567,006 | 47.39 | 93.85 | 96.31 |
| DK-1 | 23,963,165 | 7,146,744,068 | 47.72 | 93.51 | 95.52 |
| DK-2 | 23,797,007 | 7,089,937,938 | 46.77 | 93.99 | 93.73 |
| DK-3 | 21,214,319 | 6,327,563,846 | 46.78 | 93.59 | 95.17 |
| Total/Average | 200,472,117 | 59,782,912,278 | 47.28 | 93.55 | 95.39 |
